# Determining the accurate placement of a posterior portal in shoulder arthroscopy with the use of computerized tomography images

**DOI:** 10.1016/j.xrrt.2021.04.017

**Published:** 2021-05-24

**Authors:** Eilis M. Fitzgerald, Richard G. Kavanagh, Owen J. O'Connor, David I. Morrissey

**Affiliations:** a Department of Trauma and Orthopaedics, Cork University Hospital, Wilton, Cork, Ireland; b Department of Radiology, Cork University Hospital, Wilton, Cork, Ireland; c Senior Lecturer and Consultant Radiologist, Department of Radiology, Cork University Hospital, Wilton, Cork, Ireland; d Consultant Orthopaedic Surgeon, Department of Trauma and Orthopaedics, Cork University Hospital, Wilton, Cork, Ireland

**Keywords:** Arthroscopy, CT guided, posterior portal, shoulder arthroscopy

## Abstract

**Background:**

Portal placement is an important factor in performing a successful shoulder arthroscopy. Recent cadaveric studies have found variance in the anatomy of the glenohumeral joint. Our aim was to determine if computerized tomography (CT) images could be used to map the trajectory of the posterior portal objectively and then measure the distance between this trajectory and palpable landmarks to apply this knowledge to clinical practice.

**Methods:**

Two-dimensional multiplanar reformatted CT images were generated using OsiriX (Pixmeo, Switzerland) from CT images performed in a tertiary hospital over a 1-month period. The center of the glenoid was identified and a trajectory through it radiologically mapped. Horizontal and lateral measurements were taken from this trajectory to both the posterolateral edge of the acromion and tip of the coracoid.

**Results:**

Following application of inclusion and exclusion criteria, 226 shoulders were analyzed. Fifty scans were selected at random and re-reviewed by the primary examiner to assess intra-rater reliability which showed strong correlation and no significant differences between first and second measurements (*P* < .01, r > 0.6). The mean distance from acromion to portal trajectory was 1.39 cm inferiorly (95% confidence interval [CI] 1.31-1.48, standard deviation [SD] 0.65 cm) and 1.44 cm medially (95% CI 1.35-1.53, SD 0.71 cm). The mean distance from the coracoid to the trajectory was 1.71 cm inferiorly (95% CI 1.64-1.78, SD 0.55 cm) and 1.26 cm medially (95% CI 1-2-1.31, SD 0.45 cm). Paired t-test analysis between right and left shoulders within the same subject, where these data were available (n = 81), showed no significant difference (*P* > .05) between sides. Subset analysis was also performed between males and females, but only showed a significant difference between the vertical distance from the coracoid process to the center of the glenohumeral joint. This distance was shorter in females compared to males (1.56 cm in females compared to 1.84 cm in males, *P* < .001).

**Conclusions:**

Knowledge of shoulder anatomy is vital to the placement of arthroscopic portals, yet research on this topic has been based primarily on surface anatomy, small sample sized cadaveric studies or expert opinion alone. Our study shows that posterior portal placement in shoulder arthroscopy can be measured objectively using CT scanning.

Arthroscopic shoulder surgery can be technically challenging and accurate portal placement is of paramount importance for procedural success.^1^^,^^10^

Neurovascular structures travel in proximity to the joint and portal placement must be mindful of these while allowing adequate access for therapeutic procedures. Portal placement site locations are currently described in relation to surrounding palpable bony landmarks, such as the coracoid and acromion processes. Little research has been performed assessing the best site for portal placement. Most research is based on small volume cadaveric studies, anecdotal evidence, and surgeon preference.^2^, ^3^, ^4^, ^5^, ^6^^,^^8^

There are many portal placement options described in the literature,^2^, ^3^, ^4^^,^^8^, ^9^ but the posterior portal is the most commonly described. The posterior portal is traditionally introduced at a point relative to the posterolateral corner of the acromion (PCA) through an area referred to as the “soft spot”, which has now been identified as a triangular fibrocartilaginous area between the deltoid heads.^6^ More recent research has shown that using the PCA as a reference point has its own limitations due to the variability of the anatomy in this region,^5^^,^^6^ yet it still remains the standard of care across many institutions. Furthermore, the actual location of this “soft spot” varies from anywhere between 1-2 cm medial to and 2-3 cm inferior to the PCA.^12^^,^^13^

Only one paper identified by the authors assessed the possibility of using radiological data to map the procedure before surgery.^11^ This project’s main objective was to determine if a standardized method of measurement could be applied to computerized tomography (CT) images of normal shoulders to identify optimal shoulder arthroscopic portal placement. We hypothesized that this could be performed by measuring the trajectory through the center of the glenohumeral (GH) joint as a proxy for the portal position itself and then defining the distance between the trajectory and relevant palpable landmarks used in clinical practice, both anterior and posterior to the glenohumeral joint.

## Materials and methods

We performed a retrospective observational cohort study in a university teaching hospital using CT thoraces performed over a 1-month period for other clinical purposes. CT scans were identified using the hospital picture archiving and communication system (PACS) (Agfa Healthcare, Mortsel, Belgium) and performed on 64-slice CTs (General Electric, Milwaukee, WI, USA) using the following parameters: 120kV, 50-350mA, 0.625-mm slice thickness, with hybrid adaptive statistical iterative and filtered back projection reconstruction. These scans were individually reviewed to confirm the adequacy of shoulder anatomy visualization and thus suitability for inclusion in this study while simultaneously applying the below inclusion and exclusion criteria to each scan.

### Inclusion criteria

• CT thorax performed and reported on a hospital radiology system over a 1-month period
• Participants >18 years of age
• Scan including shoulder anatomy of GH joint, PCA and tip of coracoid

### Exclusion criteria

• Scan performed off site
• Participant <18 years of age
• Insufficient shoulder anatomy visible on scan
• Evidence of previous shoulder trauma for example fracture, presence of orthopedic implants, evidence of nonunion or malunion
• Excessive degenerative changes

**Figure 1:**
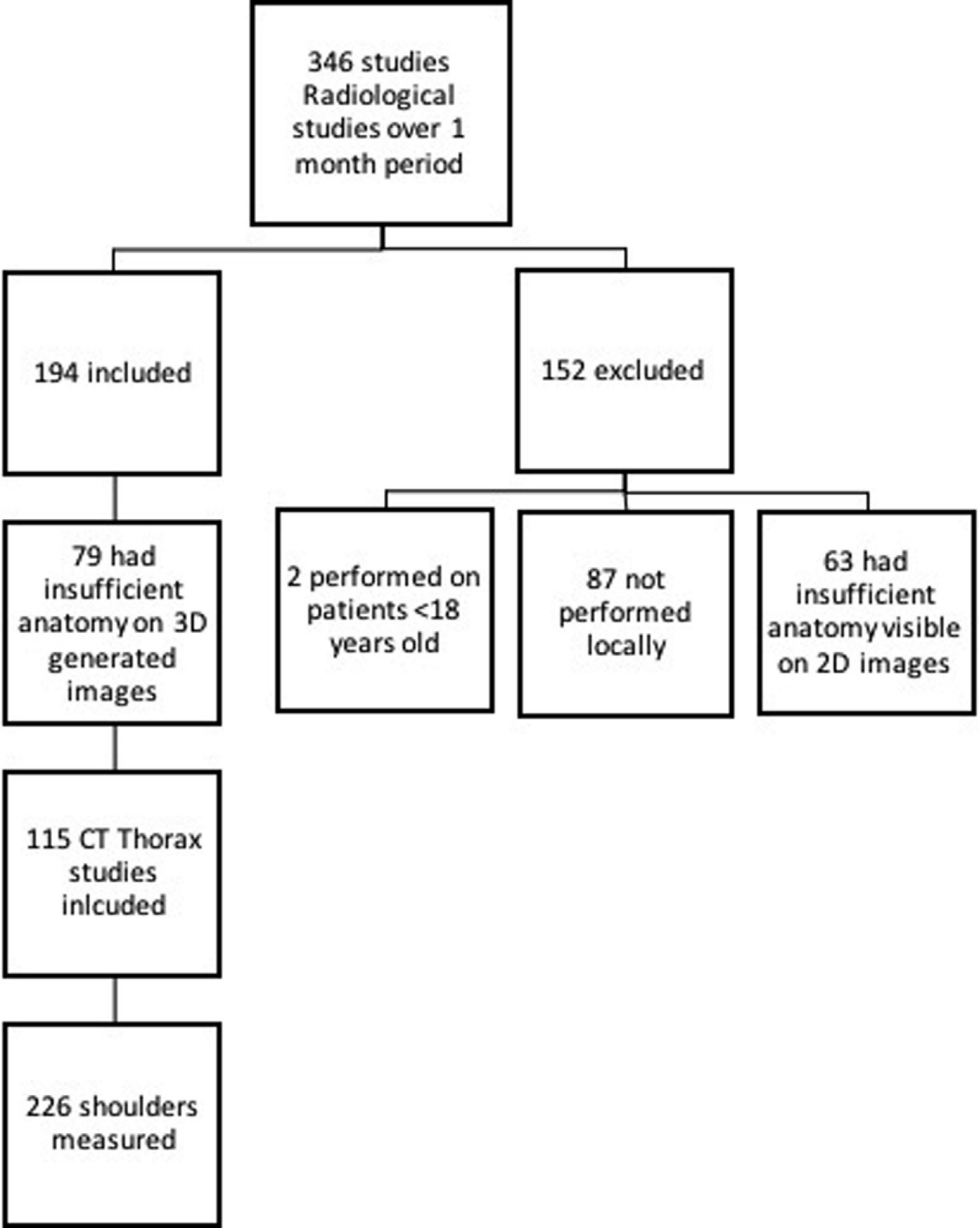
Inclusion and exclusion criteria for scan selection.

Initially 346 scans taken over a 1-month period were deemed suitable for selection. Following application of the inclusion and exclusion criteria, 194 scans were exported to the OsiriX radiological program for further inspection using its three-dimensional (3D) multiplanar reformatting and maximum intensity projection functions. Repeated assessment for anatomic depiction adequacy was performed at this stage. Review of 3D images identified a further 79 scans where shoulder anatomy was insufficiently demonstrated to facilitate anatomic assessment, leaving a study cohort of 115 scans. Four scans had only one shoulder suitable for measurement resulting in 226 shoulders identified for analysis (Fig. 1). Similar studies^7^^,^^14^ had found significance with cohorts of 120-140 shoulders and as such we felt our population would be more than large enough to determine statistical significance.

**Figure 2:**
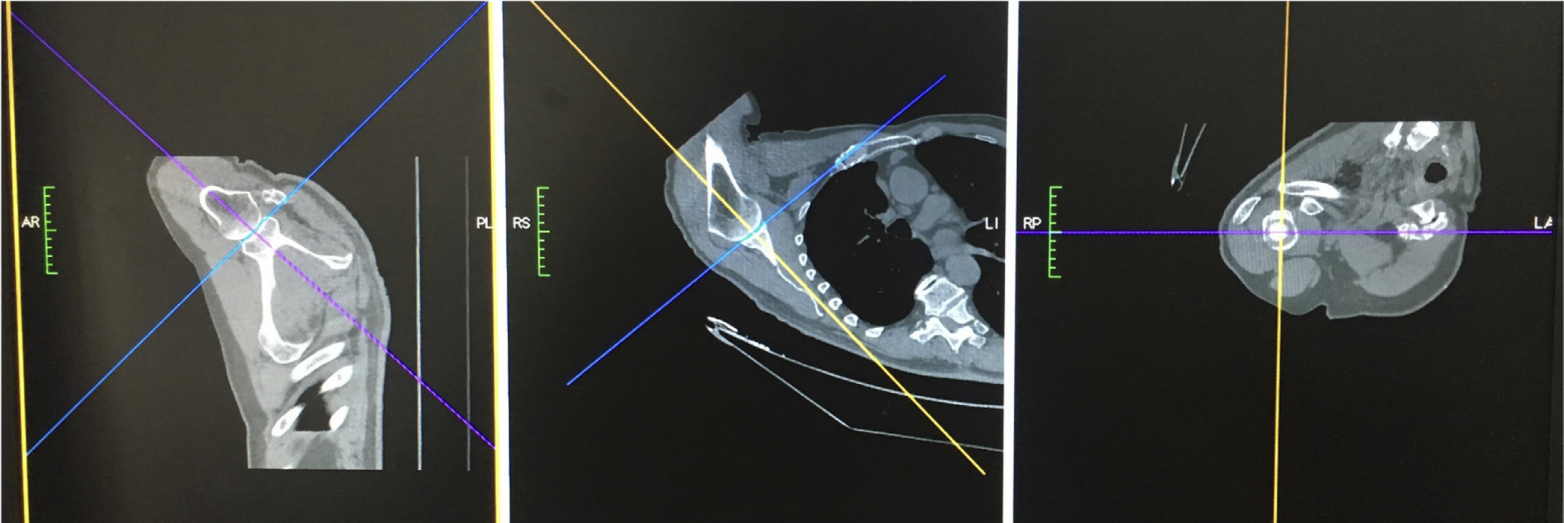
Identifying the center of the glenohumeral (GH) and the trajectories through it on 2D CT images (vertical blue, horizontal purple, reference yellow).

Measurements were made on study CTs by a single reviewer, recording up to two decimal places, using the OsiriX program. The center of the glenohumeral joint was initially defined using 2D CT images (Fig. 2). This was done by aligning the vertical axis (blue line) parallel to the glenoid surface with the orthogonal horizontal axis (purple line) traversing the center of the widest part of the glenoid. The reference axis (yellow line) was then aligned to be parallel to the scapula to allow for variable arm positions during scanning. Finally the horizontal axis position was confirmed by visualizing it intersecting the center of the glenoid itself to ensure midpoint location on 2 views.

**Figure 3:**
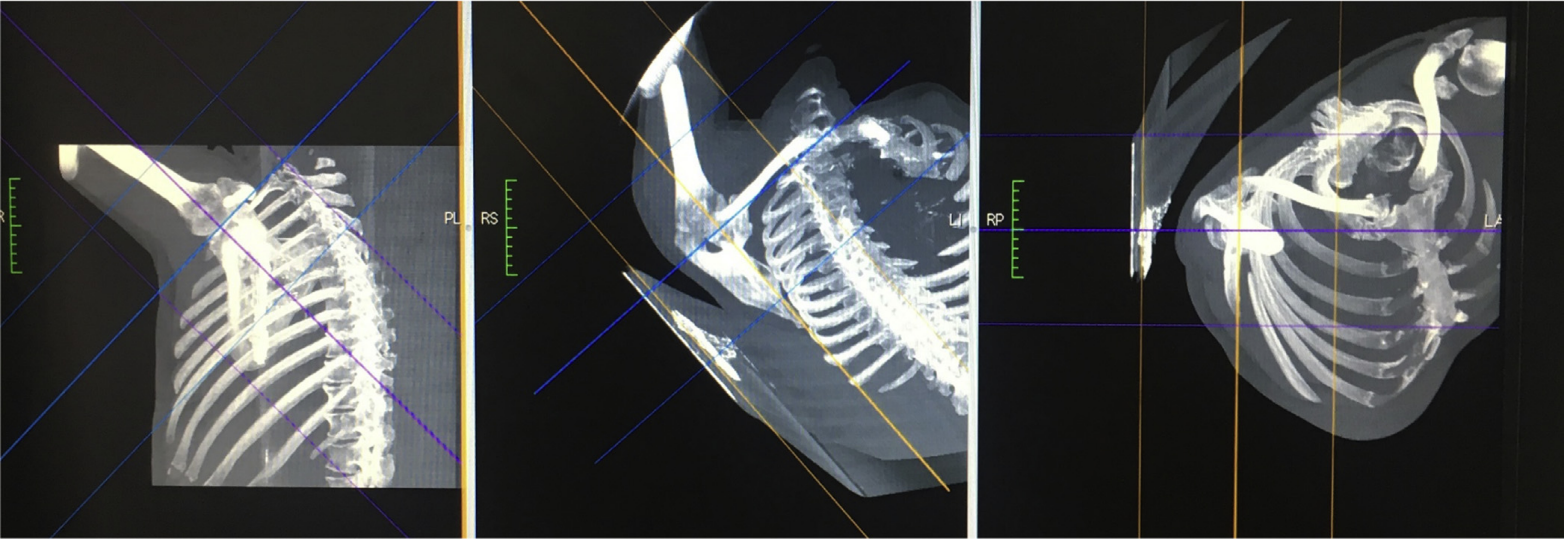
Anatomical landmarks identified using maximum intensity projection imaging system (vertical blue, horizontal purple, reference yellow).

Once the center of the joint was identified, the anatomical landmarks were identified using maximum intensity projection images (Fig. 3).

**Figure 4:**
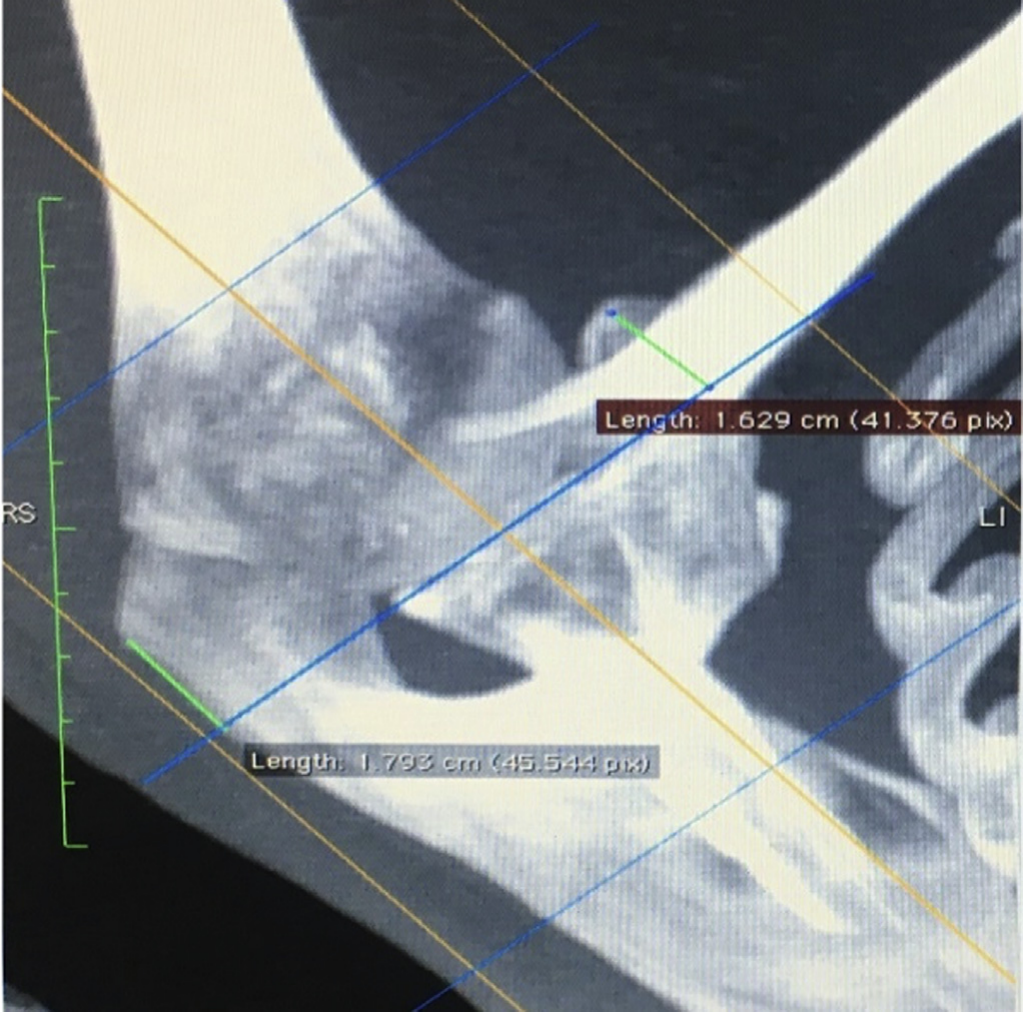
Distance between vertical axis through the center of glenohumeral (GH) joint (blue line) and palpable landmarks (posterior edge of the acromion and coracoid – green lines).

**Figure 5:**
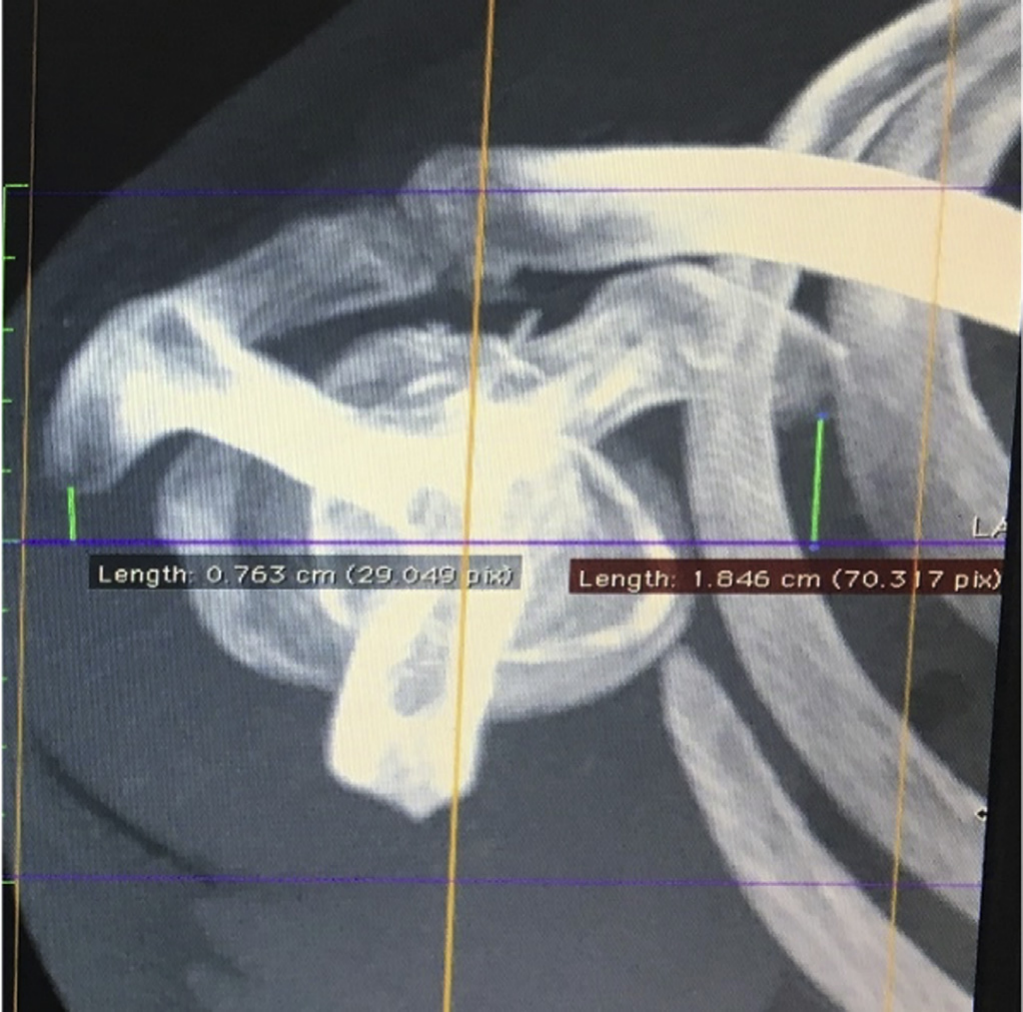
Distance between the horizontal axis through the center of glenohumeral (GH) joint (purple line) and palpable landmarks (posterior edge of the acromion and coracoid – green lines).

The distance between the posterolateral edge of the acromion and the tip of the coracoid process relative to the horizontal and vertical axes intersecting the center of the GH joint were then measured to within two decimal places (Figure 4, Figure 5).

Data were inputted into SPSS software, version 25 (IBM, Armonk, NY, USA) and tests of normality applied. Using the Kolmogorov-Smirnov test of normality, it was seen that the data were normally distributed with a *P* value of 0.200 (Table I) across all measurements. Using this information we then measured the mean, standard deviation, and confidence intervals of the data set (Table II). Paired t-test analysis was used to compare data on contralateral shoulders within the same patient (Table III). Gender analysis was performed using the independent samples t-test (Table IV).

**Table I:** Tests of normality.

	Tests of normality					
	Kolmogorov-Smirnov∗			Shapiro-Wilk		
	Statistic	df	Sig.	Statistic	Df	Sig.
Vertical acromium	.050	226	.200†	.986	226	.022
Vertical coracoid	.054	226	.200†	.994	226	.525
Horizontal acromium	.036	226	.200†	.989	226	.083
Horizontal coracoid	.041	226	.200†	.986	226	.025

∗ Lilliefors significance correction.

† This is a lower bound of the true significance.

**Table II:** Descriptive analysis.

Measure	Mean	Standard deviation	Minimum distance	Maximum distance	95% CI
Vertical distance from acromion (inferiorly)	1.39 cm	0.65 cm	−0.073 cm	3.85 cm	±0.0852 cm
Horizontal distance from acromion (medially)	1.45 cm	0.70 cm	−1.49 cm	3.5 cm	±0.0937 cm
Vertical distance from coracoid (inferiorly)	1.71 cm	0.55 cm	0.04 cm	3.31 cm	±0.0724 cm
Horizontal distance from coracoid (medially)	1.26 cm	0.45 cm	0.34 cm	2.58 cm	±0.0593 cm

**Table III:** Paired t-test for left vs right comparison (n = 81).

	Corr	Sig	Mean diff	Std Dev	Std Error mean	95% CI of the difference		t	Sig (2-tailed)
						Lower	Upper		
Vertical acromium	0.47	<0.01∗	0.08	0.68	0.08	−0.07	0.23	1.11	0.27
Vertical coracoid	0.31	0.005∗	−0.04	0.66	0.07	−0.18	0.11	−0.49	0.63
Horizontal acromium	0.69	<0.01∗	−0.07	0.51	0.06	−0.18	0.04	−1.27	0.21
Horizontal coracoid	0.42	<0.01∗	0.03	0.48	0.05	−0.07	0.14	0.62	0.53

∗ Statistically significant.

**Table IV:** Independent t-test for gender comparison.

Measurement		Levenes test		t-test for equality of means						
		F	Sig	t	df	Sig (2-tailed)	Mean difference	Std. Error difference	95% CI (lower)	95% CI (upper)
Vertical distance from acromion	Equal variances assumed	1.002	0.318	−0.34	228	0.734	−0.03	0.09	−0.2	0.14
	Equal variances not assumed			−0.34	227	0.733	−0.03	0.09	−0.2	0.14
Vertical distance from coracoid	Equal variances assumed	2.735	0.1	−3.94	228	0.000	−0.28	0.07	−0.42	−0.14
	Equal variances not assumed			−**3.94**	**227**	**<0.01**	−**0.28**	**0.07**	−**0.42**	−**0.14**
Horizontal distance from acromion	Equal variances assumed	0.004	0.951	−1.30	228	0.194	−0.12	0.09	−0.31	0.06
	Equal variances not assumed			−1.31	227	0.193	−0.12	0.09	−0.31	0.06
Horizontal distance from coracoid	Equal variances assumed	3.520	0.062	−1.37	228	0.172	−0.08	0.06	−0.2	0.04
	Equal variances not assumed			−1.37	222	0.170	−0.08	0.06	−0.2	0.04

Statistically significant outlined in bold text.

Following collection of initial data, the primary reviewer selected 50 scans at random and re-measured these to assess intra-rater reliability via the paired t-test method and correlation coefficient methods (Table V).

**Table V:** Correlation of intra-rater reliability (n = 50).

	Corr	Sig	Mean diff	Std Dev	Std Error mean	95% CI of the difference		t	Sig (2-tailed)
						Lower	Upper		
Vertical acromium	0.629	<0.01∗	0.08460	0.60527	0.08560	−0.0742	0.25662	0.988	0.328
Vertical coracoid	0.655	<0.01∗	0.09030	0.51645	0.07304	−0.05647	0.23707	1.236	0.222
Horizontal acromium	0.820	<0.01∗	0.08724	0.33118	0.04684	−0.00688	0.18136	1.863	0.069
Horizontal coracoid	0.718	<0.01∗	−0.04134	0.37272	0.005271	−0.14727	0.06459	−0.794	0.437

∗ Statistically significant.

## Results

In total, 226 shoulders were assessed on 115 patient CT scans using this technique (51% male, 49% female, mean age 69). The data for all measurements were normally distributed with a narrow range (Table I, Table II). Paired t-test analysis was used to compare measurements between right and left shoulders within the same subject and again no statistical difference between shoulders was identified, with a significant correlation between both sides on all four measurements (*P* < .05, r 0.31-0.69; Table III). Gender analysis via independent samples t-test showed no significant differences except for the vertical distance from the coracoid process to the center of the glenohumeral joint, which was shorter in females compared to males (1.56 cm in females compared to 1.84 cm in males, *P* < .001, Table IV). Intra-rater reliability was measured using paired t-test analysis and no significant difference was found between the first and second values. There was a strong, significant correlation between both measurements (*P* < .01, r > 0.6 [Table V]).

Results show that CT can be used to assess the distance between palpable landmarks, such as the PCA and the tip of the coracoid, and a trajectory which crosses the center of the glenohumeral joint. Using this data we suggest that the posterior portal is located 1.4 cm inferior to and 1.4 cm medial to the posterolateral edge of the acromion with a 95% confidence interval of ±0.085 cm and 0.09 cm, respectively. It should be aimed towards a point 1.7 cm inferior to and 1.3 cm medial to the center of the coracoid with a 95% confidence interval of ±0.07 cm and ±0.05 cm, respectively. This trajectory traverses the center of the GH joint, thus optimising the view during arthroscopy.

## Discussion

Arthroscopic shoulder surgery has become a common procedure. The glenohumeral joint is a relatively small space with overlying soft tissue and neurovascular structures. Surgery can therefore be technically challenging and potentially dangerous if carried out with suboptimal access. Current practice regarding the technique of portal placement is based on anecdotal, personal or low volume cadaveric evidence.^7^ Our aim was to assess the accuracy of current practice from an objective radiographic standpoint.

Traditionally the posterior portal is introduced via a “soft spot” which has been described in the literature as anywhere from 1-2 cm medial to and 2-3 cm inferior to the posterolateral corner of the acromion.^11^, ^12^ Our results describe this point more definitively and provide an accurate range in a large cohort of patients.

Although our data have a relatively narrow range (Table III), it highlights the fact that one size may not fit all. This novel measurement technique allows for preoperative planning of individualized portal placement to aid in complex procedures.

This project is unique in so far as we previously identified only one other paper which sought to objectify portal placement using radiological data.^7^ The use of radiological data in planning orthopedic operations is not a novel one. Radiological templating in arthroplasty is now done routinely in most centers and navigational systems are evolving in elective orthopedic subspecialties. There is little data present for arthroscopic surgery however. A CT technique was used in the current study; however, these methods could be applied to 3D MRI sequences in a similar manner. MRI is routinely performed prior to shoulder arthroscopy and there is no radiation burden for patients.

This study involves a large cohort of patients, objectively measured using a freely available radiological program. Other studies published on the topic of portal placement have mainly been of small sample sizes. We had a relatively even male to female split and measured both left and right shoulders.

The use of CT scanning and measurement is objective and we found it to have good intra-rater reliability. Using the scapula as a reference trajectory allows any CT to be used as it accounts for varying shoulder position during scanning. The data set produced showed little variance in the measurements obtained and good correlation with clinical practice. One limitation of the study is that the present values have not been correlated with *in vivo* results, which represents a potential avenue of future research. This could initially be performed in cadavers with imaging prior to shoulder arthroscopy. This would allow assessment of the iatrogenic risk profile of this trajectory to be identified after the procedure, an issue not dealt within the current study.

## Conclusions

This is the first large cohort study to radiologically assess prospective portal position in shoulder arthroscopy. These initial findings corroborate current practice and offer an objective method for individualized measurements which could serve as a model for preoperative assessment methods.

## Disclaimers

Funding: No funding was obtained for this project.

Conflicts of interest: The authors, their immediate family, and any research foundation with which they are affiliated did not receive any financial payments or other benefits from any commercial entity related to the subject of this article.

## References

[1] Berjano P. (1998). Complications in arthroscopic shoulder surgery. Arthroscopy.

[2] Davidson P.A., Rivenburgh D.W. (2002). The 7-o'clock posteroinferior portal for shoulder arthroscopy. Am J Sports Med.

[3] Davidson P.A., Tibone J.E. (1995). Anterior-inferior (5 o'clock) portal for shoulder arthroscopy. Arthroscopy.

[4] Difelice G.S., Williams R.J., Cohen M.S., Warren R.F. (2001). The accessory posterior portal for shoulder arthroscopy: description of technique and cadaveric study. Arthroscopy.

[5] Edelson J.G., Taitz C. (1993). Bony anatomy of coracoacromial arch: implications for arthroscopic portal placement in the shoulder. Arthroscopy.

[6] Ercakmak B., Gunenc Beser C., Ozsoy M.H., Demiryurek M.D., Bayramoglu A., Hayran K.M. (2018). Soft spot: the important zone at the standard posterior portal of shoulder arthroscopy. Turk J Med Sci.

[7] Gauci M.O., Deransart P., Chaoui J., Urvoy M., Athwal G.S., Sanchez-Sotelo J. (2020 Dec). Three-dimensional geometry of the normal shoulder: a software analysis. J Shoulder Elbow Surg.

[8] Goubier J.N., Iserin A., Augereau B. (2001). The posterolateral portal: A new approach for shoulder arthroscopy. Arthroscopy.

[9] Huri G., Üzümcügil A., Biçer O.S., Ozturk H., McFarland E.G., Doral M.N. (2015). An alternative endoscopic portal for suprascapular nerve approach: an anatomic study. Knee Surg Sports Traumatol Arthrosc.

[10] Ishida Y., Chosa E., Taniguchi N. (2015). Pseudoaneurysm as a complication of shoulder arthroscopy. Knee Surg Sports Traumatol Arthrosc.

[11] Ito Y., Nakao Y., Manaka T., Naka Y., Matsumoto I., Takaoka K. (2008). Advantages of a navigation system to create portals for shoulder arthroscopy: a preliminary investigation. Curr Orthopaedic Pract.

[12] Kalairajah Y., Tennent T.D. (2005). The 'hiss sign': an effective way of confirming satisfactory intra-articular placement of the first portal in shoulder arthroscopy. Ann R Coll Surg Engl.

[13] Neviaser T.J. (1987). Arthroscopy of the shoulder. Orthop Clin North Am.

[14] Totlis T., Natsis K., Pantelidis P., Paraskevas G., Iosifidis M., Kyriakidis A. (2014). Reliability of the posterolateral corner of the acromion as a landmark for the posterior arthroscopic portal of the shoulder. J Shoulder Elbow Surg.

